# Validity and Reliability of a Smartphone-Based Gait Assessment in Measuring Temporal Gait Parameters: Challenges and Recommendations

**DOI:** 10.3390/bios15070397

**Published:** 2025-06-20

**Authors:** Sam Guoshi Liang, Ho Yin Chung, Ka Wing Chu, Yuk Hong Gao, Fong Ying Lau, Wolfe Ixin Lai, Gabriel Ching-Hang Fong, Patrick Wai-Hang Kwong, Freddy Man Hin Lam

**Affiliations:** Department of Rehabilitation Sciences, The Hong Kong Polytechnic University, 11 Yuk Choi Road, Hung Hom, Kowloon City, Hong Kong

**Keywords:** gait, sensor, accelerometer, wearable, smartphone, temporal parameters

## Abstract

Smartphone-embedded inertia sensors are widely available nowadays. We have developed a smartphone application that could assess temporal gait characteristics using the built-in inertia measurement unit with the aim of enabling mass screening for gait abnormality. This study aimed to examine the test–retest reliability and concurrent validity of the smartphone-based gait assessment in assessing temporal gait parameters in level-ground walking. Twenty-six healthy young adults (mean age: 20.8 ± 0.7) were recruited. Participants walked at their comfortable pace on a 10 m pathway repetitively in two walking sessions. Gait data were simultaneously collected by the smartphone application and a VICON system during the walk. Gait events of heel strike and toes off were detected from the sensors signal by a peak detection algorithm. Further gait parameters were calculated and compared between the two systems. Pearson Product–Moment Correlation was used to evaluate the concurrent validity of both systems. Test–retest reliability was examined by the intraclass correlation coefficients (ICCs) between measurements from two sessions scheduled one to four weeks apart. The validity of smartphone-based gait assessment was moderate to excellent for parameters involving only heel strike detection (r = 0.628–0.977), poor to moderate for parameters involving detection of both heel strike and toes off (r = 0.098–0.704), and poor for the proportion of gait phases within a gait cycle. Reliability was good to fair for heel strike-related parameters (ICC = 0.845–0.388), good to moderate for heel strike and toes-off-related parameters (ICC = 0.827–0.582), and moderate to fair for proportional parameters. Validity was adversely affected when toe off was involved in the calculation, when there was an insufficient number of effective steps taken, or when calculating sub-phases with short duration. The use of smartphone-based gait assessment is recommended in calculating step time and stride time, and we suggest collecting no less than 100 steps per leg during clinical application for better validity and reliability.

## 1. Introduction

Level walking is one of the fundamental activities of daily living which depends on the functioning of the complicated, multi-level locomotion system. Normal walking involves the contribution of the higher center of the brain, cerebellum, proprioceptive receptors, motor control of muscles, joint movements, and the adaptation of the skeletal system [1]. Impairment in such may produce an abnormal gait, or even severely hinder our mobility at home and in the community, hence adversely affecting social participation and quality of life [2,3].

While the extent and nature of gait abnormality vary in different medical conditions, clinical gait analysis as a diagnostic test may enhance patient management by identifying the details of gait deficits, hence contributing to treatment decision-making and patient outcomes in different patient populations [4,5,6,7,8]. For example, accurate detection of gait impairments could help to identify the early stage of cognitive decline with a slow gait speed [9,10], evaluate the severity of motor impairment and adapt treatment recommendations in children with cerebral palsy [11,12], and predict prospective falls in older adults with an area under curve value of 0.82 when combined with other traditional screen tests [13].

Time-based quantitative gait assessment and balance evaluation are common to measure physical function, such as timed-up-and-go test and gait speed [14]. However, these assessments do not evaluate the quality of movement and fail to provide details of gait deficit. On the other hand, qualitative gait assessments are usually carried out by physiotherapists using scoring scales, such as the Dynamic Gait Index [15]. However, the accuracy of such a method is questionable as it depends on the subjective judgment of the assessor [16]. Other instrumental methods for measuring gait parameters with higher accuracy and objectiveness, including force-sensitive plates or sensors and optoelectronic stereophotogrammetry [17], are available in clinical and laboratory settings. Yet, those gait analysis tools may be hardly applicable in the healthcare system, due to the high cost, low portability, labor-intensive operation, and time-consuming procedures. Introducing a convenient and easy-to-use gait assessment tool can be a new option for community-based gait and balance assessment, allowing patients to self-monitor their own performances, as well as sharing timely data with caregivers, hence further utilize the anomaly-detecting and treatment-planning function of gait assessment in the community. Therefore, a cost-effective, portable, easy-to-use, and accurate gait assessment method is urged to be developed and deployed to cope with the surging demand of the overstretched public healthcare system in a few decades.

Under technological advancement, the development of wearable sensors for health data collection has surged recently [18,19,20]. One of the major domains is the use of portable devices for gait analysis. Inertial sensors, which derive acceleration and angular velocity signals, can provide real-time gait information with a lower cost, higher practicality, and fewer restraints on the testing environments, giving them great potential to replace traditional quantitative gait analysis tools in specialized centers [21]. Previous studies have investigated the effectiveness of wearable accelerometers in gait assessment [22,23,24,25]. However, multiple sensors were placed on the lower body, including the foot, shank, and thigh, which would interfere with natural walking and limit its application to the daily life of individuals [26]. A single sensor on the lower trunk can be an alternative to such methods in measuring gait events to further simplify its application [27,28]. Furthermore, the single sensor attached at the waist showed the best reliability and validity compared to other placement locations [29]. It was also the most common location used in gait analysis in the scientific community [30,31,32].

Smartphones, which have built-in micro-electro-mechanical sensors (MEMSs) like accelerometers and gyroscopes, could be a solution to developing a gait assessment tool with high accessibility and massive popularity among citizens. By sensing the linear and rotational orientation of the phone, the data of the embedded sensors could be translated into various gait parameters for measuring body motion [33,34]. With sensors embedded in portable devices like smartphones, motion-tracking gait analysis would be more cost-effective, convenient, and user-friendly. Even when the feasibility of measuring gait parameters with an accelerometer placed on the lower trunk during walking has been well established by Zijlstra and Hof [35], previous works have rarely employed such a method with a single sensor or smartphone. More often, multiple sensors and smartphones were used to improve the validity of different walking outcomes generated [36,37]. However, this would limit its applicability in the community [38,39].

Therefore, the present study aimed to investigate whether a single smartphone-embedded accelerometer on the lower trunk can capture valid temporal gait parameters, including sub-phase duration within the gait cycle in level walking, and establish the reliability of the smartphone-based gait assessment. The target variables of our smartphone application include stride time, swing phase duration, and double support duration, which are all relevant indicators of pathological gait [40] and predictors of fall [41]. This study is expected to contribute to the development of a potential gait assessment tool that is convenient, highly accessible, and affordable in different settings, thus enhancing health monitoring and clinical application.

## 2. Materials and Methods

### 2.1. Participants

Twenty-six healthy adults were recruited from the Hong Kong Polytechnic University. To be included in the study, the participants have to be healthy adults without specific conditions that affect the gait pattern. Exclusion criteria of participants were (1) an existing or a history of musculoskeletal, peripheral, or central nervous system condition(s) that limit their independent ambulation, (2) a history of psychiatric illness or neurological disorder that affects their compliance, and (3) skin diseases which hinders the attachment of reflective markers on their skin.

All participants joined this study voluntarily and were briefed about their right to withdraw from the study. Ethics approval for this study was granted by the authority of the Human Subjects Ethics Sub-committee of The Hong Kong Polytechnic University (HSEAR20230726003) and informed consent was obtained from all participants.

### 2.2. Experimental Procedure

The data collection procedure was conducted at the Gait and Motion Analysis Laboratory of the Hong Kong Polytechnic University. Participants attended two assessment sessions that lasted for approximately one hour each.

Participants were asked to wear comfortable and well-fitting outfits with their usual footwear for outdoor walking in both data collection sessions. This attire could facilitate the usual gait pattern and accurate placement of VICON markers on their bodies. Loose-fitting clothes will be secured with cloth tape. Any reflective material on the clothing or footwear was taped to prevent interference with the VICON motion capturing system.

A 10 m path was used for the walking trials, and 150 total steps were expected to be obtained from each subject to calculate an accurate clinical result. Each subject was asked to walk in a straight line from one point to another at their normal speed following the tester’s instructions (Supplementary Materials File S1, Figure S1). Each subject would take 30 trials to capture the expected step counts. Gait parameters during the walk were captured by the VICON system and the smartphone system simultaneously. An Arduino-produced analogue signal was sent to the VICON system from the smartphone via Bluetooth to synchronize the start time of both systems.

### 2.3. VICON Motion Capture System

To capture and measure the kinematic data of participants in gait, a sixteen-camera 3D motion capture system (VICON Nexus 2, VICON NexusTM, VICON Motion System Ltd., Oxford, UK) was used. As a well-established tool for gait analysis, the VICON motion capture system has demonstrated high accuracy and reliability in previous studies and was used as the criterion measure for gait analysis [42,43,44]. Therefore, it is considered to be a ‘gold standard’ for gait analysis.

To record the kinematic characteristics, a system with sixteen VICON cameras in the laboratory was calibrated in accordance with the standardized protocol from VICON at the beginning of each data collection session. Thirty-nine pearl hard reflective markers with a diameter of 14 mm were then attached to the anatomical landmarks of a participant according to the plug-in gait full body model for 3D movement tracking and analysis during walking (Supplementary Materials File S1, Figure S2) [45]. All data collected would be used to reconstruct motion for gait analysis.

### 2.4. Smartphone-Based Gait Assessment

**Figure 1:**
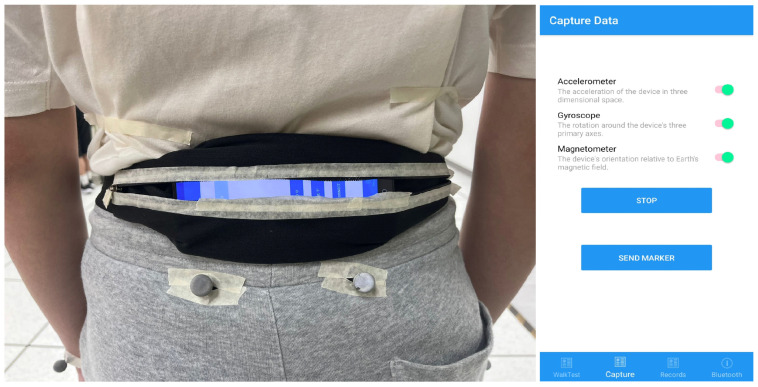
Smartphone placement and orientation with the running custom application interface. While clicking the ‘START’ button, the application will continuously collect the data of acceleration and gyroscope until the ‘STOP’ button is clicked. The application will send a signal to the VICON motion capture system for synchronization upon clicking the ‘SEND MARKER’ button.

The model of the smartphone used in this study was the Samsung Galaxy S22 (SM-S9010, Samsung Electronics, Suwon, Republic of Korean). The smartphone with built-in sensors including accelerometer and gyroscope (LSM6DSO, STMicroelectronics, Plan-les-Ouates, Switzerland) was placed horizontally on the waist of the participants at the level of L4–L5 with the screen facing outward, and the top edge (front camera) oriented toward the participant’s left side. A semi-elastic belt that wrapped snugly around the waist was used to attach the smartphone to the lower back (Figure 1). The adjustable sliders of the belt were tensioned to ensure the secure fixation of the smartphone. The acceleration and gyroscope data were collected during walking in three orthogonal anatomical axes, i.e., the anterior-posterior, mediolateral, and vertical axes, using a custom application. The application originally requested the data from sensors in over 400 Hz sampling frequency and downsampled it to 100 Hz for calculation efficiency. The sensor precision is 0.002 m/s^2^ with a range of ±78.453 m/s^2^ for the accelerometer and 0.0006 rad/s with a range of ±17.453 rad/s for the gyroscope.

In order to address the test–retest reliability of the smartphone-based accelerometer, each participant was asked to attend another session within a month and at least a week after their first session. The protocol and task conditions were kept identical to the first session.

### 2.5. Data Processing

#### 2.5.1. VICON Data

All the marker trajectories were labeled using the software of VICON Nexus 2, and processed with the full-body plugin gait pipelines. The gait events (i.e., the instant of heel-strike and toe off) of the footsteps that fell on the force plate in the middle of the path were automatically detected by VICON Nexus 2. The gait events of the rest footsteps during the walking trial were generated using the analysis function of “Autocorrelate Events”. Further temporal parameters related to the walking trial were calculated using the generated CSV file with the gait events data.

#### 2.5.2. Smartphone Data

**Figure 2:**
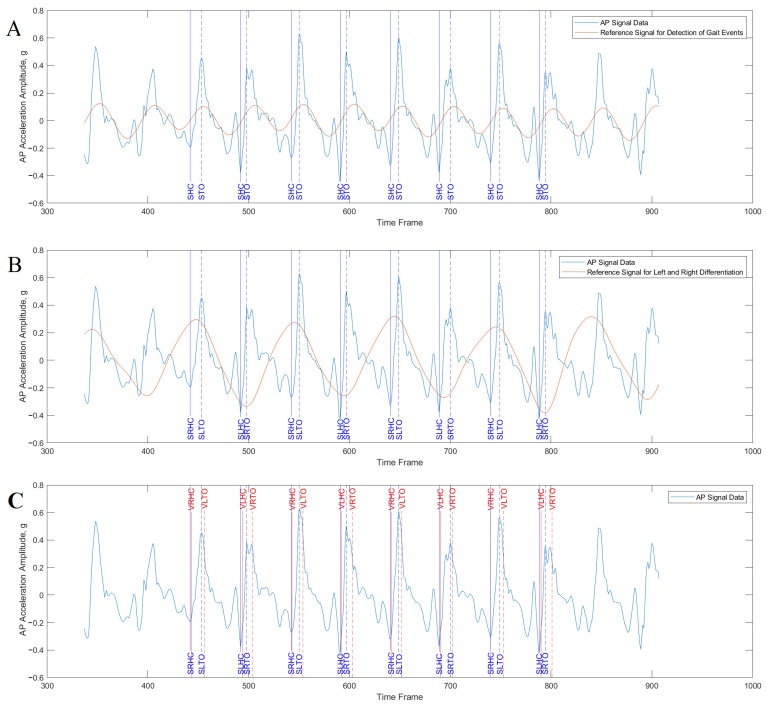
An example of the data processing of the smartphone data for gait events. (**A**) Gait events including the heel contact (heel strike) and toes off were defined based on the reference signal. (**B**) Left and right were detected based on the reference gyroscope signal. (**C**) Compare the results of smartphone with the gold standard of the VICON motion capture system. AP, anterio-posterior. SHC, smartphone heel contacts. STO, smartphone toes off. SRHC, smartphone right heel contacts. SLHC, smartphone left heel contacts. SRTO, smartphone right toes off, SLTO, smartphone left toes off, VRHC, VICON right heel contact, VLHC, VICON left heel contact, VRTO, VICON right toes off. VLTO, VICON left toes off.

To extract the data, acceleration and gyroscope signals collected by the smartphone application were uploaded to the computer and processed in the MATLAB software (R2021a, Mathworks, Natick, MA, USA). The initial and last three steps of each trial were excluded from the analysis to eliminate the acceleration and deceleration phases during gait initiation and termination. The gait events of heel-strike and toes off of each foot were then identified by the method validated in a standalone sensor [28,46]. This algorithm was adopted as it was the few open-source algorithms that estimate heel strike and toes off based on waist acceleration. This method is also suitable for smartphones with lower processing powers, compared with other algorithms that require more complicated computation [47]. During the data processing, a smoothed anterior–posterior acceleration signal was first generated by using a Butterworth filter (4th order, cut-off frequency 2 Hz). Using the minimum peaks of the smoothed signal as the reference, the minimum peaks of the raw anterior–posterior signal that are nearest to the reference minimum peaks are defined as the gait events of the heel strike [28]. The maximum peaks of the raw anterior–posterior acceleration signal that are the closest to the maximum peaks of the reference signals are defined as the gait events of the toes off [46]. Secondly, the gyroscope data of the anterior–posterior axis was used to define the left or right step for the detected gait events of the heel strike. The raw gyroscope data were filtered by a Butterworth filter (4th order, cut-off frequency 2 Hz), which generated a smooth signal showing the pelvic rotation movement during walking. The heel strikes falling on the positive threshold value are defined as right heel strikes while the ones on the negative threshold value are left heel strikes. The gait events detection procedure is shown in Figure 2. The identification of the heel strike and toes off allowed the calculation of temporal gait parameters (i.e., step time, stride time, stance duration, swing duration, single/double support duration, Table 1).

#### 2.5.3. Generating Temporal Parameters

**Figure 3:**
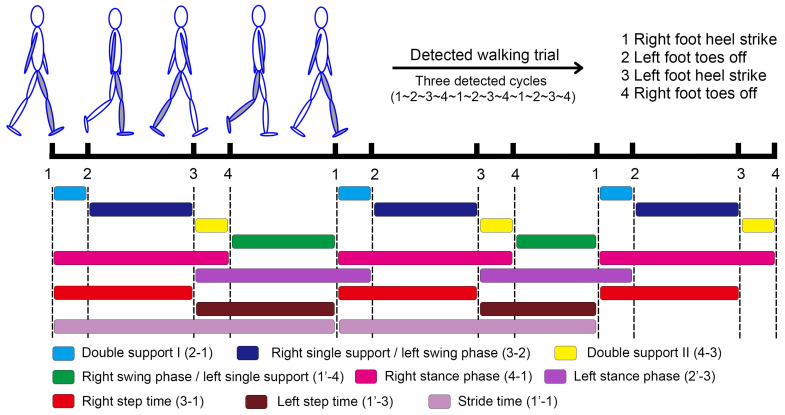
Gait events and gait parameters identification and definition. The comma superscript represents the gait event of the next gait cycle.

The gait events generated from the VICON system and smartphone were first labeled as specific numbers for further calculation in MATLAB software. Right foot heel contact, left foot toes off, left foot heel contact, and right foot toes off were labeled as “1”, “2”, “3”, and “4”, respectively. All the walking trials would start at a detected right foot heel contact and end with the right foot toes off (Figure 3). This alignment method labeled the gait events in an orderly time series and enhanced computational efficiency, but it resulted in the number of left steps being more than the number of right steps, as the first step is always initiated by the left foot. Temporal parameters, including the duration of double support I, right foot single support/left foot swing phase, double support II, right foot swing phase/left foot single support, right stance phase, step time, stride time, and the proportion of these sub-phases would be generated for all the walking trials. Mean values were used to represent the gait characteristics of each participant. Proportional parameters were calculated based on the above values (Table 1).

### 2.6. Statistical Analysis

Statistical analysis was performed using SPSS statistical software (IBM^®^, SPSS^®^ Statistics, Version 25). The normality of the data was verified using the Shapiro–Wilk test. Means and standard deviations were calculated for each parameter for both systems over all the walking trials.

To examine the validity, the Pearson Product–Moment Correlation was used to compare the data captured by the VICON system and the smartphone, examining the correlation between both methods. For non-parametric parameters, Spearman Rank Correlation was used instead of Pearson Product–Moment Correlation. Validities of parameters were classified as excellent (r > 0.90), good (0.75 < r ≤ 0.90), moderate (0.50 < r ≤ 0.75), fair (0.25 < r ≤ 0.50), or poor (r ≤ 0.25 or *p* > 0.05) based on their correlation coefficients [48]. Bland–Altman plots with 95% limits of agreement were generated to determine the degree of agreement between the two systems. Percentage bias was calculated based on the Bland–Altman plots with VICON as reference [49].

To examine the test–retest reliability of the accelerometer, the intraclass correlation coefficients (ICC_3, 1_) between measurements from the two sessions were calculated. Reliabilities of parameters were classified as excellent (ICC > 0.90), good (0.75 < ICC ≤ 0.90), moderate (0.50 < ICC ≤ 0.75), fair (0.25 < ICC ≤ 0.50), or poor (ICC ≤ 0.25 or *p* > 0.05) based on their ICC value [50]. Statistical significance (*p*-value) for all tests was determined at the level of 0.05 [51].

## 3. Results

**Figure 4:**
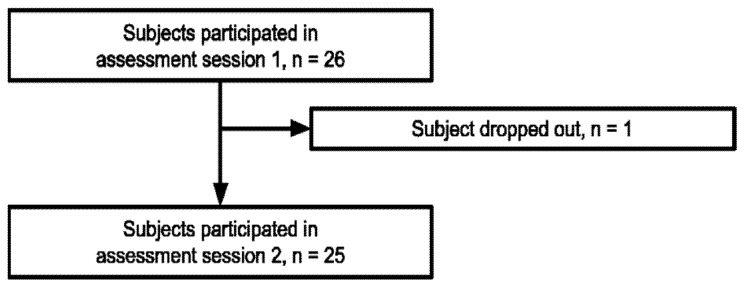
Flow diagram of study subjects.

In total, 26 participants completed the first session of assessment for validity (Figure 4; 13 male, 13 female, age = 20.8 ± 0.7 years). In total, 1 subject dropped out of the second session and 25 participants completed the second session of assessment for reliability (Table 2; 13 male, 12 female, age = 20.8 ± 0.7 years).

### 3.1. Validity

The Pearson Product–Moment Correlation coefficient (*r*), bias, percentage bias, and limits of agreement for each parameter are listed in Table 3. Generally, the *r* values for the measured gait parameters displayed a wide range of variability.

Duration parameters derived from the heel strike only displayed excellent to moderate validity. Step time of both legs and stride time demonstrated excellent validity (*r* = 0.977, *p* < 0.001; *r* = 0.969, *p* < 0.001) while left and right step times demonstrated moderate validity (*r* = 0.628, *p* < 0.001; *r* = 0.553, *p* = 0.003).

Duration parameters derived from heel strike and toe off displayed moderate to poor validity. Right and left stance phases and right single support demonstrated moderate validity (*r* = 0.704, *p* < 0.001; *r* = 0.554, *p* = 0.003; *r* = 0.461, *p* = 0.018) while the right swing phase demonstrated fair validity (*r* = 0.467, *p* = 0.016). Double support I and II demonstrated poor validity (*r* = 0.098, *p* = 0.634; *r* = 0.387, *p* = 0.051). All proportion parameters demonstrated poor validity (*r* = 0.091–0.350, *p* > 0.05).

**Figure 5:**
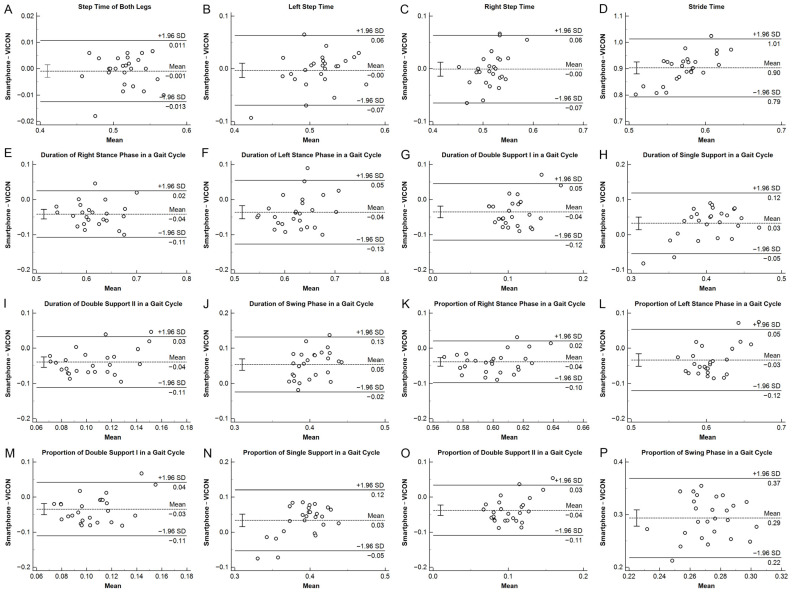
Bland–Altman plots of different gait parameters between VICON and smartphone. (**A**) Step Time of Both Legs; (**B**) Left Step Time; (**C**) Right Step Time; (**D**) Stride Time; (**E**) Duration of Right Stance Phase in a Gait Cycle; (**F**) Duration of Left Stance Phase in a Gait Cycle; (**G**) Duration of Double Support I in a Gait Cycle; (**H**) Duration of Single Support in a Gait Cycle; (**I**) Duration of Double Support II in a Gait Cycle; (**J**) Duration of Swing Phase in a Gait Cycle; (**K**) Proportion of Right Stance Phase in a Gait Cycle; (**L**) Proportion of Left Stance Phase in a Gait Cycle; (**M**) Proportion of Double Support I in a Gait Cycle; (**N**) Proportion of Single Support in a Gait Cycle; (**O**) Proportion of Double Support II in a Gait Cycle; (**P**) Proportion of Swing Phase in a Gait Cycle. Note: Differences between VICON and smartphone (smartphone—VICON) are plotted against means between VICON and smartphone for different gait parameters. Dashed lines represent the means of difference between VICON and smartphone while solid lines represent the limits of agreement (mean ± 1.96 SD). Error bars represent 95% confidence intervals of the means of difference between VICON and smartphone.

The biases of duration parameters derived from heel strike only were close to zero (bias = −0.001~−0.004) (Table 3). Meanwhile, the biases of other gait parameters were comparatively greater with a magnitude of −0.042–0.038 s. Among the 16 gait parameters, step time of both legs, left and right step time, and stride time showed small limits of agreement while other parameters showed larger limits of agreement (Table 3). Detailed Bland–Altman plots are available in Figure 5.

### 3.2. Reliability

Generally, the test–retest reliabilities of the smartphone gait analysis system for different parameters were good to fair (Table 4).

Duration parameters derived from heel strike only displayed good to fair reliability. Step time of both legs and stride time demonstrated good reliability (ICC = 0.845, *p* < 0.001; ICC = 0.829, *p* < 0.001). Left step time demonstrated moderate reliability (ICC = 0.684, *p* < 0.001) while right step time demonstrated fair reliability (ICC = 0.388, *p* = 0.028).

Duration parameters derived from heel strike and toes off displayed good to moderate reliability. The right stance phase and right single support demonstrated good reliability (ICC = 0.796, *p* < 0.001; ICC = 0.827, *p* < 0.001) while the left stance phase, double support I and II, and right swing phase demonstrated moderate reliability (ICC = 0.691, *p* < 0.001; ICC = 0.709, *p* < 0.001; ICC = 0.615, *p* < 0.001; ICC = 0.582, *p* = 0.001).

Proportion parameters displayed moderate to fair reliability. Left stance phase, double support I and II, and single support demonstrated moderate reliability (ICC = 0.710, *p* < 0.001; ICC = 0.681, *p* < 0.001; ICC = 0.628, *p* < 0.001; ICC = 0.710, *p* < 0.001) while right stance phase and swing phase demonstrated fair reliability (ICC = 0.429, *p* = 0.016; ICC = 0.429, *p* = 0.016).

## 4. Discussion

This study aimed to investigate the validity and reliability of a smartphone-embedded accelerometer on the lower trunk in providing temporal parameters in level walking. The results obtained showed good to moderate validity in gait parameters derived from heel strike events only (left step time, right step time, stride time, and step time of both legs), and a moderate to fair validity in gait parameters derived from heel strike and toes off events (swing phase, single support, left stance phase and right stance phase). The validity of double support I, double support II, and all proportion parameters were poor. The test–retest reliabilities of the smartphone gait analysis system for different parameters varied from good to fair. In general, the better the validity of the gait parameter calculated, the better the reliability.

### 4.1. Inaccuracy in Toes-off-Derived Gait Parameters

Our results showed that gait parameters derived from heel strike and toes off together were less accurate than those derived from heel strike only (Table 3). The inaccuracy in toes-off-derived gait parameters may be due to less accurate detection of toes off events by smartphone. With reference to VICON, we observed larger errors of the smartphone in detecting toes off (−0.047 to −0.043 s) than heel strikes (−0.011 to −0.012 s) in our sample (Supplementary Materials File S1, Table S1).

Incorrect detection of toes off events by smartphone may be due to difficulty in toes off event identification from accelerometer signal. In the anteroposterior accelerometer signal (Supplementary Materials File S1, Figures S3 and S4), multiple peaks were noted around the time of toes off events, leading to the difficulty in identifying the exact time when toes off happened. The current method identifies the maximum peak as the toes off event [46], causing potential errors in some participants. Regarding the amplitude of the troughs (heel strike) and peaks (toes off) in the accelerometer data, heel strike events had a larger magnitude of change than toe off, indicating that toe off is a softer and more subtle event, making it more difficult to detect accurately. Another study also demonstrated difficulty in identifying toes off events using an accelerometer-based sensor with a different algorithm [52]. Such difficulty in toes off detection might explain why gait analysis research studies often exclude toes off in their calculation of gait parameters.

Other potential reasons causing the inaccuracy in toe off detection include synchronization error, slow walking speed [46], and flat-footed landing during walking. However, given a similar level of systematic error was not noted on the detection of heel strike, synchronization error should not be the main reason for the inaccuracy. In addition, given the average walking speed of the young participants was 1.5 m/s^2^, which is relatively high, no evidence supports the inaccuracy derived from slow gait speed and gait pattern. Future studies should focus on modifying the accelerometer calculation algorithm to improve the validity of toe off event identification. For example, a specific pattern wave or a specific open-source AI filter box may be used to filter the raw data to correct the early error peaks mentioned above to improve the accuracy of toes off detection.

### 4.2. A Higher Step Count Is Associated with Better Validity

Within duration parameters derived from heel strike only, our results showed that the step time of both legs was more accurate than the isolated left or right step time. Since all of those parameters do not involve toes off in their calculations, the difference in validity cannot be explained by inaccuracy in toes off identification. Instead, better accuracy of the step time of both legs over isolated left or right step time may be due to the larger number of steps collected for the step time of both legs. To further evaluate this observation, we divided the participants into three groups using the number of valid steps detected. Subgroup analyses were performed on the participants with the highest number of steps (n = 9) and those with the lowest number of steps (n = 9), respectively. Participants with more steps consistently report better correlation with VICON in step time (average step count = 109–247, r = 0.802–0.989), compared with those with the lowest number of steps (average step count = 43.4–114.7, r = 0.584–0.972) (Supplementary Materials File S1, Tables S2 and S3). It appears that the number of effective steps has to exceed 100 for better validity.

### 4.3. Gait Phases with Shorter Durations Is Associated with Worse Validity

Within duration parameters derived from heel strike and toes off, the study showed a wide range of discrepancies in the results of validity (Table 3). Since all of those parameters included one toes off event in their calculation, the error in toe off identification alone cannot explain such a difference in results. In addition, the mean number of samples used to calculate those parameters was consistent, which denied the possibility of a difference in the number of samples affecting the validity. The difference in duration of each gait phase can serve as a possible explanation. As shown in Table 3, gait parameters with longer durations showed better validity. Gait phases with shorter duration would contain a larger proportion of errors yielded in heel strike and toe off detection, leading to poorer validity.

### 4.4. Inaccuracy in Proportion Parameters

Generally, the validity and reliability of proportional parameters were poorer than those of duration parameters of the same gait phases (Table 3 and Table 4). Poorer validity and reliability in proportion parameters can be attributed to the complexity of their calculation. This was because the calculation of proportion parameters was carried out by dividing the duration parameters of gait phases by the stride time. This introduced errors from both duration parameters and stride time to proportion parameters, leading to an increase in the inaccuracy of proportion parameters compared to duration parameters.

### 4.5. Clinical Implications and Future Direction

To be used for clinical application, an outcome measure has to be both valid and reliable. Out of the 16 gait parameters that we have measured, high validity and good test–retest reliability were observed in step time and stride time, which were events derived from heel strikes and of longer duration. Therefore, our research supports the use of our smartphone-based gait analysis system in assessing step and strike time.

Isolated left and right step time demonstrated moderate validity and moderate to fair reliability. Since their difference in validity was only contributed by the fewer number of effective steps, we categorize their applicability in clinical use as acceptable, but only under the circumstance that a sufficient number of effective steps (above 100 steps in each leg) were taken into account when calculating these parameters.

For other parameters, their validity ranged from moderate to poor due to their nature of gait characteristics (toes off-involved events, events with short duration) or their complexity in calculation (proportional parameters). Further refinement of the data processing algorithm is required to improve their validity before clinical application.

Our study has set a foundation for developing gait assessments using a smartphone system. Our results can also guide future studies related to smartphone gait analysis to refine the protocol for better results. Future studies should be conducted to improve the accelerometer calculation algorithm to address the systemic error in toes off identification. Further exploration of the cut-off value of effective steps taken to calculate a valid result can be carried out to improve the clinical utility of the smartphone system.

### 4.6. Limitations

This study exhibited several limitations. First, due to the set configuration of the VICON system, the study was constrained to an indoor laboratory setting which would confine the number of steps in each trial. Thus, the natural variability of walking gait could not be reflected. In addition, as only level walking at a preferred speed was evaluated, faster motion, turning, or other movements of daily locomotion were not assessed. To further improve the validity of the smartphone system in gait analysis, future studies should include more continuous steps in a single trial to assess the variability of gait and compare the results on different walking styles or environments.

In terms of generalizability, our participants were limited to healthy young adults with normal gait patterns and did not include populations in other age groups or populations with pathological gait characteristics. Therefore, further validation of the smartphone system is required with different age groups, ethnicities, and populations with pathological gait or motion impairment whose gait pattern displays a wider range of variations to improve the external validity of smartphones as a gait analysis tool.

Additionally, only one smartphone model was used in our study. Previous studies investigating the feasibility of smartphone-based gait assessment varied in smartphone models used, such as IOS-based and Android-based smartphones [53]. Differences in smartphone models or smartphone applications may influence the validity and reliability of the gait parameter calculated. Since the smartphone model used by individuals may vary among the general population, future studies should investigate the differences between the gait parameters calculated by different smartphone models.

Furthermore, the reliability and validity presented depend highly on the algorithm used to detect heel strikes and toes off. The results of the study could only be generalized to the algorithm adopted.

Lastly, we did not quantify the damping characteristics of the belt which might partly contribute to the inaccuracy. However, we tried our best to secure the smartphone on the lower back of the participants during the assessment without causing discomfort. We believe our experimental setup could reflect the use of smartphones in assessing gait characteristics in real-life applications.

## 5. Conclusions

This study demonstrated the feasibility of using a smartphone for gait assessment in young healthy adults. Sixteen clinically relevant gait parameters were calculated from identifying heel strike and toe off events. Step time of both legs and stride time demonstrated excellent validity and good reliability. Right stance phase, left stance phase and single support showed moderate validity and good to moderate reliability. Left step time and right step time demonstrated moderate validity and moderate to fair reliability. These gait parameters show potential clinical applicability in gait assessment, but only when a sufficient number of effective steps (above 100 steps in each leg) were collected for calculating these parameters. For other parameters, their validity ranged from moderate to poor due to their nature of gait characteristics (toe off-involved events, events with short duration) or their complexity in the calculation (proportional parameters). Overall, this study lays the foundation for the future development of smartphone-based gait analysis technology, thus enhancing community-based healthcare service.

## Figures and Tables

**Table 1:** Definition and derivation of duration parameters.

Parameter	Definition
Parameters Derived from the Gait Events of Heel Strike	
Step time of both legs	Mean time between two consecutive heel strikes of reciprocal legs for each subject.
Left step time	Mean time from a right heel strike to the next left heel strike for each subject.
Right step time	Mean time from a left heel strike to the next right heel strike for each subject.
Stride time	Mean time between two consecutive right heel strikes for each subject.
Parameters Derived from the Gait Events of Heel Strike and Toes Off	
Right stance phase duration	Mean time from a right heel strike to the following right toe off for each subject.
Left stance phase duration	Mean time from a left heel strike to the following left toes off for each subject.
Double support I duration	Mean time from a right heel strike to the following left toe off for each subject.
Right single support/Left swing phase duration	Mean time from a left toe off to the following left heel strike for each subject.
Double support II duration	Mean time from a left heel strike to the following right toe off for each subject.
Right swing phase/Left single support duration	Mean time from a right toe off to the following right heel strike for each subject.
Proportion Parameters	
Right stance phase proportion	Right stance phase duration divided by stride time
Left stance phase proportion	Left stance phase duration divided by stride time
Double support I proportion	Double support I duration divided by stride time
Right single support/Left swing phase proportion	Right single support/Left swing phase duration divided by stride time
Double support II proportion	Double support II duration divided by stride time
Right swing phase/Left single support proportion	Right swing phase/Left single support duration divided by stride time

**Table 2:** Subject characteristics.

Demographics	Assessment Session 1	Assessment Session 2
Number of subjects	26	25
Male (%)	13 (50%)	13 (52%)
Age (years)	20.8 ± 0.7	20.8 ± 0.7
Height (cm)	168.8 ± 8.5	169.2 ± 8.5
Weight (kg)	62.6 ± 9.9	63.1 ± 9.7
Gait speed (m/s)	1.50 ± 0.12	1.50 ± 0.12
Average number of steps	177.3 ± 66.3	177.8 ± 67.6

**Table 3:** Validity of gait parameters between VICON and smartphone.

Parameter	Average Step Count	VICON Mean ± SD (n = 26)	Smartphone Mean ± SD (n = 26)	r	Bias	Percentage Bias	Lower LOA	Upper LOA
Duration parameters derived from HS only (sec)								
Step time of both legs	177.3	0.516 ± 0.028	0.515 ± 0.028	0.977 **	−0.001	−0.2%	−0.013	0.011
Left step time	103.2	0.513 ± 0.032	0.510 ± 0.043	0.628 **	−0.003	−0.6%	−0.069	0.063
Right step time	74.1	0.516 ± 0.025	0.518 ± 0.039	0.553 *	−0.001	−0.2%	−0.065	0.063
Stride time	74.1	1.028 ± 0.054	1.025 ± 0.054	0.969 **	−0.004	−0.4%	−0.030	0.023
Duration parameters derived from HS and TO (sec)								
Right stance phase	103.2	0.637 ± 0.045	0.595 ± 0.043	0.704 **	−0.042 **	−6.5%	−0.108	0.025
Left stance phase	74.1	0.642 ± 0.043	0.606 ± 0.053	0.554 *	−0.036 **	−5.6%	−0.127	0.055
Double support I	103.2	0.127 ± 0.026	0.090 ± 0.035	0.098 ‡	−0.035 **	−27.7%	−0.116	0.045
Right single support/Left swing phase	103.2	0.388 ± 0.030	0.419 ± 0.050	0.568 * ‡	0.032 *	8.3%	−0.055	0.119
Double support II	103.2	0.125 ± 0.025	0.087 ± 0.036	0.387 ‡	−0.039 **	−31.3%	−0.112	0.033
Right swing/Left single support phase	74.1	0.391 ± 0.025	0.429 ± 0.034	0.467 *	0.038 **	9.8%	−0.024	0.100
Proportion parameters								
Right stance phase	103.2	0.620 ± 0.020	0.581 ± 0.026	0.244 ‡	−0.039 **	−6.2%	−0.098	0.021
Left stance phase	74.1	0.624 ± 0.024	0.591 ± 0.043	0.310 ‡	−0.033 **	−5.3%	−0.120	0.054
Double support I	103.2	0.123 ± 0.023	0.087 ± 0.033	0.091 ‡	−0.034 **	−27.8%	−0.111	0.042
Right single support/Left swing phase	103.2	0.376 ± 0.023	0.408 ± 0.044	0.255 ‡	0.034 **	9.0%	−0.053	0.120
Double support II	103.2	0.121 ± 0.022	0.085 ± 0.036	0.350 ‡	−0.037 **	−31.0%	−0.109	0.034
Right swing/Left single support phase	74.1	0.380 ± 0.020	0.419 ± 0.026	0.244 ‡	0.039 **	10.2%	−0.021	0.098

*Note.* HS = heel strike, TO = toe off, r = Pearson correlation coefficient unless specified, bias = mean difference (smartphone—VICON), percentage bias = bias/VICON mean, LOA = limit of agreement. * *p* < 0.05. ** *p* < 0.001. ‡ Spearman rank correlation coefficient is tested instead because the parameter is not normally distributed or cannot assume normal distribution.

**Table 4:** Reliability of gait parameters between two sessions of smartphone.

Parameter	Average Step Count	Session 1 Smartphone Mean ± SD (n = 25)	Session 2 Smartphone Mean ± SD (n = 25)	ICC_3, 1_
Duration parameters derived from HS only (sec)				
Step time of both legs	177.8	0.516 ± 0.028	0.518 ± 0.037	0.845 **
Left step time	103.4	0.509 ± 0.044	0.519 ± 0.039	0.684 **
Right step time	74.4	0.518 ± 0.036	0.518 ± 0.048	0.388 *
Stride time	74.4	1.027 ± 0.054	1.038 ± 0.077	0.829 **
Duration parameters derived from HS and TO (sec)				
Right stance phase	103.4	0.596 ± 0.044	0.604 ± 0.048	0.796 **
Left stance phase	74.4	0.608 ± 0.053	0.608 ± 0.063	0.691 **
Double support I	103.4	0.090 ± 0.035	0.089 ± 0.031	0.709 **
Right single support/Left swing phase	103.4	0.419 ± 0.050	0.430 ± 0.043	0.827 **
Double support II	103.4	0.087 ± 0.036	0.085 ± 0.030	0.615 **
Right swing/Left single support phase	74.4	0.431 ± 0.033	0.433 ± 0.043	0.582 *
Proportion parameters				
Right stance phase	103.4	0.580 ± 0.026	0.582 ± 0.024	0.429 *
Left stance phase	74.4	0.592 ± 0.044	0.585 ± 0.034	0.710 **
Double support I	103.4	0.087 ± 0.033	0.085 ± 0.028	0.681 **
Right single support/Left swing phase	103.4	0.408 ± 0.044	0.415 ± 0.034	0.710 **
Double support II	103.4	0.085 ± 0.036	0.082 ± 0.028	0.628 **
Right swing/Left single support phase	74.4	0.420 ± 0.026	0.418 ± 0.024	0.429 *

*Note.* HS = heel strike, TO = toe off, ICC = intraclass correlation coefficient. * *p* < 0.05. ** *p* < 0.001.

## Data Availability

All the data can be accessed by contacting the correspondence author.

## Supplementary Materials

The following supporting information can be downloaded at: https://www.mdpi.com/article/10.3390/bios15070397/s1. Figure S1. Setting of the laboratory and walking path for walking trials. Figure S2. VICON markers set configuration on a subject. Figure S3. A raw anterior-posterior acceleration signal over time collected by the smartphone. Figure S4. A raw anterior-posterior acceleration signal over time collected by the smartphone. Table S1. Errors Between VICON and Smartphone in Heel strike and Toes off Time. Table S2. Validity of Gait Parameters in Subgroup with Nine Participants with the Largest Number of Steps. Table S3. Validity of Gait Parameters in Subgroup with Nine Participants with the Smallest Number of Steps.
