# Supplementary material for: Validity and Reliability of a Smartphone-Based Gait Assessment in Measuring Temporal Gait Parameters: Challenges and Recommendations
Source: Biosensors (Basel). 2025 Jun 20;15(7):397. doi: 10.3390/bios15070397 (PMC12294008; doi:10.3390/bios15070397)
Supplement: Supplementary file 1 [file biosensors-15-00397-s001.zip › biosensors-3650080-supplementary.pdf]

Supplemental materials. S1

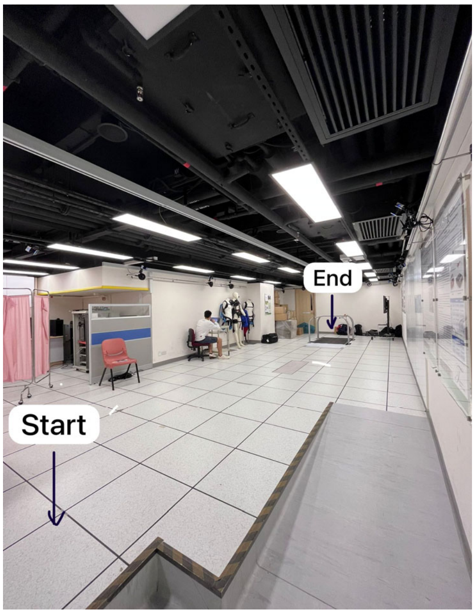

Figure S1. Setting of the laboratory and walking path for walking trials. The force plate of the VICON motion capture system was located at the middle of the walking path. The participants walk from the "Start"/"End" position to the "End"/"Start" position with their normal walking speed.

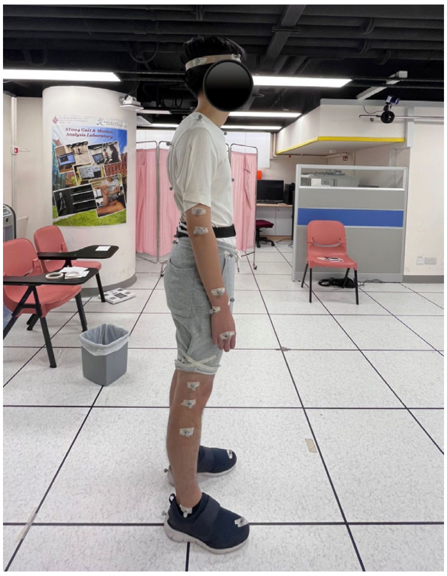

Figure S2. VICON markers set configuration on a subject. The markers were placed on head (the left and right temple, and the both side of back head), trunk (the cervical vertebra of 7th, 10th, calvicle, sternum, and right back surface of scapula), upper limbs (both sides of the acromio-clavicular joint, the upper lateral 1/3 surface of the arm, the epicondyle, the lower lateral 1/3 surface of the forearm, the thumb side of the wrist joint, the 5th metacarpal side of the wrist joint and the distal end of the third metacarpal), pelvis (both sides of the anterior and posterior superior iliac), and lower limbs (both sides of the lower lateral 1/3 surface of the thigh, the lateral of knee joint axis, the lower 1/3 surface of the tibia, the lateral malleolus, the posterior of calcaneous, and the distal of second metatarsal head).

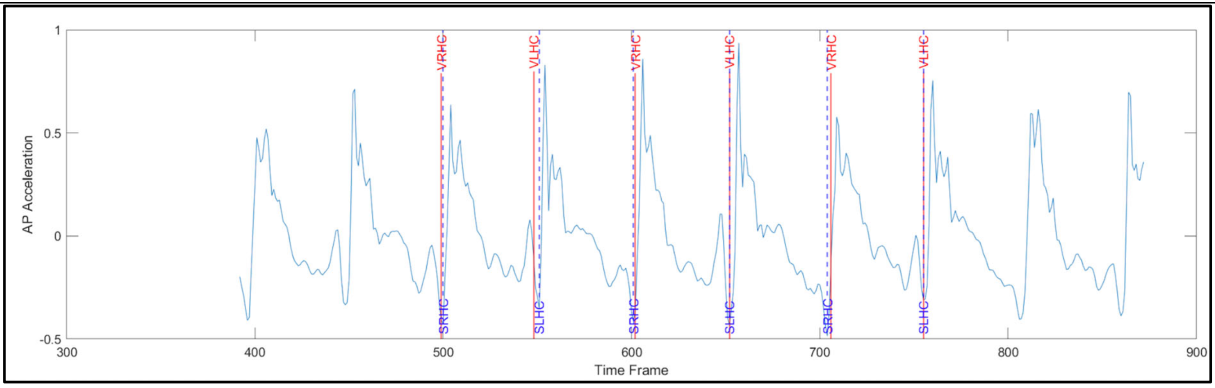

Figure S3. A raw anterior-posterior acceleration signal over time collected by the smartphone. Red solid lines represent heel strikes detected by VICON and blue dashed lines represent heel strikes detected by smartphone. SRHC, Smartphone right heel contact. VRHC, Vicon right heel contact. SLHC, Smartphone left heel contact. VLHC, Vicon left heel contact.

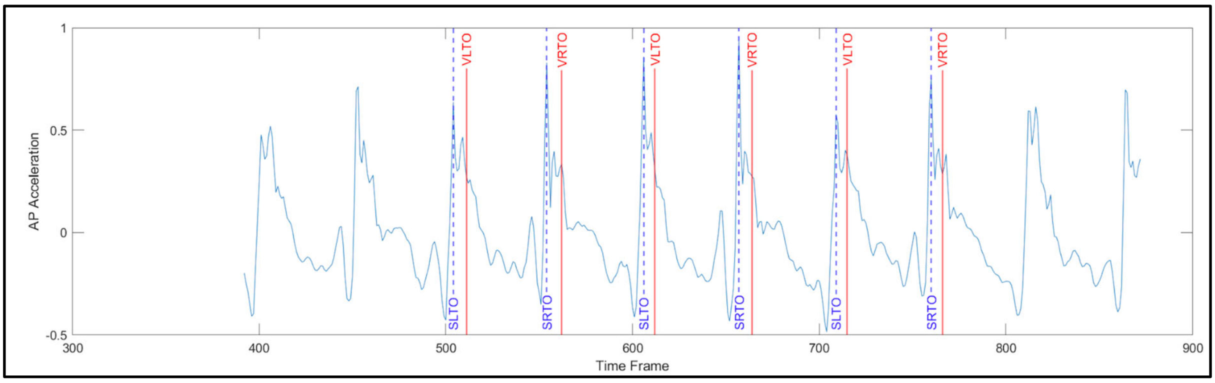

Figure S4. A raw anterior-posterior acceleration signal over time collected by the smartphone. Red solid lines represent toe off detected by VICON and blue dashed lines represent toe off detected by smartphone. SLTO, Smart left toes off. VLTO, Vion left toes off. SRTO, Smartphone tight toes off. VRTO, Vicon right toes off.

Table S1. Errors Between VICON and Smartphone in Heel strike and Toes off Time

Gait Event	mean ± SD
Errors in heel strikes (sec)	
Both legs	−0.011 ± 0.026
Left leg only	−0.011 ± 0.034
Right leg only	−0.012 ± 0.023
Errors in toe off (sec)	
Both legs	−0.045 ± 0.015
Left leg only	−0.043 ± 0.018
Right leg only	−0.047 ± 0.015

Note. Errors = time difference between Smartphone and VICON in gait events (Smartphone − VICON). The above table summarized errors between VICON and smartphone in heel strikes and toe-off time. Generally, the smartphone system detected gait events earlier than VICON and errors in toe off were larger than that in heel strike.

Table S2. Validity of Gait Parameters in Subgroup with Nine Participants with the Largest Number of Steps

Parameter	n	r
Step time of both legs	247.0	0.989**
Left step time	137.8	0.905**
Right step time	109.2	0.802**

Note. n = average number of samples per subject, r = Pearson correlation coefficient.
** $p < 0.001$.

Table S3. Validity of Gait Parameters in Subgroup with Nine Participants with the Smallest Number of Steps

Parameter	Average step count	r
Step time of both legs	114.7	0.972**
Left step time	71.2	0.728*†
Right step time	43.4	0.584

Note. n = average number of samples per subject, r = Pearson correlation coefficient unless specified.
* $p < 0.05$.
** $p < 0.001$.
† Spearman rank correlation coefficient is tested instead because the parameter is not normally distributed.
